# Come for Information, Stay for Support: Harnessing the Power of Online Health Communities for Social Connectedness during the COVID-19 Pandemic

**DOI:** 10.3390/ijerph182312743

**Published:** 2021-12-03

**Authors:** Brian M. Green, Casey A. Hribar, Sara Hayes, Amrita Bhowmick, Leslie Beth Herbert

**Affiliations:** 1 Health Union, LLC, Philadelphia, PA 19107, USA; sara.hayes@health-union.com (S.H.); amrita.bhowmick@health-union.com (A.B.); LB.herbert@health-union.com (L.B.H.); 2 School of Medicine, University of North Carolina at Chapel Hill, Chapel Hill, NC 27599, USA; casey_hribar@med.unc.edu; 3 Department of Health Behavior, Gillings School of Global Public Health, University of North Carolina at Chapel Hill, Chapel Hill, NC 27599, USA

**Keywords:** eHealth, chronic disease, online community, social support, COVID-19

## Abstract

The COVID-19 pandemic created a globally shared stressor that saw a rise in the emphasis on mental and emotional wellbeing. However, historically, these topics were not openly discussed, leaving those struggling without professional support. One powerful tool to bridge the gap and facilitate connectedness during times of isolation is online health communities (OHCs). This study surveyed Health Union OHC members during the pandemic to determine the degree of COVID-19 concern, social isolation, and mental health distress they are facing, as well as to assess where they are receiving information about COVID-19 and what sources of support they desire. The survey was completed in six independent waves between March 2020 and April 2021, and garnered 10,177 total responses. In the United States, OHCs were utilized significantly more during peak lockdown times, and the desire for emotional and/or mental health support increased over time. Open-ended responses demonstrated a strong desire for connection and validation, which are quintessential characteristics of OHCs. Through active moderation utilizing trained moderators, OHCs can provide a powerful, intermediate and safe space where conversations about mental and emotional wellbeing can be normalized and those in need are encouraged to seek additional assistance from healthcare professionals if warranted.

## 1. Introduction

Although social isolation, loneliness, and mental health distress have always been commonplace, societal stigma often means they are not openly discussed [1]. However, the COVID-19 pandemic created a globally shared stressor that saw a rise in the emphasis on mental and emotional wellbeing. From popular icons such as Michelle Obama freely speaking about experiencing “low-grade depression” as a result of the pandemic and societal unrest in America [2], to the World Health Organization’s emphasis on increased mental health infrastructure due to an impending and critical increase in demand [3], the COVID-19 pandemic has changed the way we talk about, prioritize, and consume healthcare and services that address mental health needs.

Despite still being in the midst of the pandemic, research so far points toward COVID-19′s ubiquitous negative impact on mental health [3,4,5,6]. A Kaiser Family Foundation survey from April to May of 2021 found that 30% of adults in the United States (U.S.) reported symptoms of depression or anxiety, a rise of more than 10% higher than expected based on pre-pandemic trends. In addition, nearly a quarter of those with symptoms of psychological distress who reported needing mental health services were not able to access them [7]. These COVID-19 impacts are far-reaching; however, factors such as a previous lack of social support, perceived likelihood of survival, comorbid mental or physical health conditions, and challenging home demands, such as homeschooling young children, are all contributors to an increased risk for mental health distress [4,6].

In research conducted prior to the pandemic, social connectedness and social support were associated with benefits such as decreased risk of cardiovascular disease and depression, improved immune system functioning, and reduced morbidity and mortality, among others [8,9,10,11].

A metanalysis of 40 studies confirmed that comorbidities of mental health and chronic physical conditions are a burden for people, not just in the developed world where research often focuses, but also in developing and emerging countries [12]. While psychological interventions of various types can improve a patient’s quality of life [13,14,15], there are mixed results depending upon in-patient or out-patient settings [16].

A positive state of mind as a result of strong social support, is also linked to greater medication adherence, a critical concern when it comes to living with one or more chronic health conditions [17]. 

Chronic health conditions are of particular interest in examining societal responses to the COVID-19 pandemic, as physical lockdowns and fears of contagion prevented the ease of access to in-person healthcare [18]. In addition, current research still suggests that those with pre-existing chronic conditions (including those with a history of malignancy) have a greater risk of developing severe COVID-19 and accompanying significant morbidity and mortality than those without [19,20]. Further, the population of chronically ill individuals tends to be older, and inherently possesses an increased susceptibility to social isolation and deteriorating mental health [21].

Especially in the context of the COVID-19 pandemic, telemedicine provides a means for patients to seek care for a variety of concerns in a convenient, accessible, and safe manner. While telemedicine for mental health is on the rise, there are still questions regarding patient willingness to embrace these new options when discussing sensitive issues. Additional concerns include payor issues with telemedicine services and the equitable access to technology required in order to undergo sessions [22,23,24]. 

One powerful tool to bridge the gap and facilitate social support and connectedness during times of isolation is online health communities (OHCs). OHCs are online platforms in which individuals with similar health conditions or experiences can share information, support, and connections [25,26]. OHCs provide a temporally flexible space that allows people to connect across geographic locations and often with anonymity [26,27]. OHCs allow patients to play as active or passive a role as they would like, while helping with identity development, self-confidence, personal validation, social interconnectedness, navigation of complex emotions, and fostering a sense of purpose [27,28,29,30]. Although OHCs are not a platform for medical care nor advice, they can provide an environment to gauge similar experiences and gain confidence to seek professional support. 

In order to facilitate safe, online spaces that provide support, social connection, and information, Health Union created an Adaptive Engagement Model for OHCs [30]. At the beginning of the pandemic, Health Union had 28 active OHCs. Each community is a separate URL that matches the name of the condition, for example, Lupus.net, and has social media pages on Facebook, Instagram, and Twitter.

These OHCs are contextual and situational, and feature three core structural elements, including social support, adaptive engagement, and active moderation. Similar to physical communities, they rely on a shared identity (connection to a specific chronic health condition), social norms, and commonly an experience of societal stigma. These features, taken together in the creation and subsequent management and moderation of the OHCs, are used to facilitate the sharing of relevant health information (or content), build relationships, and harness participant interdependence through passive and active engagement opportunities [30]. Given the Adaptive Engagement Model’s high emphasis on combating social isolation and creating space for normalizing conversations about life with a chronic illness, including mental health impacts, these OHCs may serve as a stepping stone for intermediate support as people struggle with considering and initiating professional services for mental health or emotional support.

The present study was conducted primarily to determine the information needs and supportive resources needed by people with chronic health conditions from Health Union’s 28 online communities during the pandemic. After seeing how community members continued to engage and support each other throughout the pandemic and become less interested in specific content regarding COVID, the authors decided to conduct a secondary analysis of these data to further study social connectedness.

The aim of this study was to survey current Health Union OHC members to determine the degree of COVID-19 concern, social isolation, and mental health distress they are facing, as well as assessing where they are receiving information about COVID-19 and what sources of support they require. Although other research focused on the mental and emotional impacts of COVID-19 and the use of telemedicine or other technologies to access healthcare services, our efforts aim to investigate the novel space in between, wherein OHCs may play a critical role in fostering wellbeing, especially when access to traditional in-person services may be limited.

## 2. Materials and Methods

### 2.1. Data Collection and Survey Design

As this was a secondary analysis of a de-identified data set originally collected for quality improvement purposes, it was considered exempt from human subject review. This research was conducted according to the guidelines of the Declaration of Helsinki and other relevant laws in the U.S. Informed consent was obtained from all survey participants via an introductory email, allowing people to voluntarily click through to accept or decline the invitation to complete the Qualtrics survey. Survey responses were anonymous and not linked to individual identifiers. All email or IP addresses were stripped from the data set prior to data cleaning, storage, and access of the final data set for analysis by the authors.

Data were collected in an online survey format hosted through Qualtrics Survey Software (Qualtrics International Inc., Seattle, WA, USA). The survey was administered in six waves between 19 March 2020 and 19 April 2021. The first survey wave was sent to members of 10 separate OHCs hosted, managed, and moderated by Health Union. Subsequent waves were expanded to eventually reach a total of 28 separate OHCs managed and moderated by Health Union. Each platform provides a space for support, engagement, and education around the chronic health condition of focus [30]. In total, the survey was sent to community members via email across the six waves. Banners and other site features advertising the survey were also displayed on each platform during data collection periods. Data collection for each wave lasted between three and eight days, tailored with the aim of securing an adequate sample size, while being inclusive of people with many different chronic health conditions (Table 1).

In order to participate in the survey, respondents needed to be at least 18 years old, live in the United States, have a chronic health condition from a pre-specified list (full list in Supplementary Materials), and be aware of COVID-19. Survey questions focused on life with a chronic condition during the pandemic. Initial topics of interest included current treatments used to manage chronic health conditions, where information was obtained, and concern regarding COVID-19. In addition, questions regarding changes in personal health behaviors as a result of the pandemic, changes in established treatment plans, desired support sources, communication with healthcare providers, and financial impacts were also included.

Given the evolving and uncertain nature of the COVID-19 pandemic, it was necessary to adjust topics slightly to most accurately reflect the current status of the pandemic for that time period. For example, waves three through six included questions about telehealth at the same time that telehealth was expanded by executive order through the Centers for Medicare and Medicaid Services during this time period [31]. Waves five and six included questions surrounding pandemic burnout, quality of life, and vaccination status. Due to this required flexibility, each wave ranged from 35 to 41 questions. 

Participants who provided complete responses to the survey were entered in a drawing to receive a U.S. e-gift card for each wave. The drawing for the first wave featured USD 50 gift cards, while waves two through six featured USD 25 gift cards.

### 2.2. Measures

#### 2.2.1. Perception of COVID-19

Concern for COVID-19 was assessed in each wave through the question, “At this time, how concerned do you feel about the novel coronavirus (COVID-19)?” Concern was measured on a 7-point Likert scale (1 = Not at all concerned to 7 = Very concerned). Wave six (post-vaccine availability) had a slightly modified version of this question by gauging agreement with the statement, “I am still very concerned about my risk of contracting COVID-19” (7-point Likert scale with 1 = Strongly disagree and 7 = Strongly agree). In a similar fashion, participants were asked about their concern with COVID-19 in the context of having a chronic health condition in each survey wave (Supplementary Materials). Significant concern was a response of 6 or 7, while a lack of concern was a response of 1 or 2. In several waves (waves two, three, and four), participants were also asked in an open-ended question to provide one word that described how they were feeling about COVID-19.

#### 2.2.2. Utilized and Desired Sources of COVID-19 Information and Support

In order to determine current COVID-19 information resource use, respondents were prompted in waves one through four with the question, “What sources are you using to learn more about the novel coronavirus (COVID-19)?” Participants were prompted to select as many as applied from a list of resources, including social networking sites, Internet search engines, government websites, online blogs or support communities, and TV news reports (Supplementary Materials). Subsequently, participants were asked, “What types of information and/or support would be most helpful to you right now?” Respondents were permitted to choose up to three desired types of information or support including information from their healthcare provider about COVID-19 in relation to their health condition, emotional and/or mental health support, financial support for medications, and home delivery options (Supplementary Materials).

#### 2.2.3. Quality of Life and Health-Related Behavioral Changes

Later waves, specifically waves five and six, incorporated questions about quality of life, mental health impacts, burnout, and perception of returning to pre-pandemic “normal” life. Changes in more tangible behaviors were asked in wave five, through a 3-option ranking of doing less, doing the same as, or doing more of a specific behavior pre-pandemic versus present. For example, participants were asked to rank their current social media use with pre-pandemic levels on this scale. Less tangible changes, such as stress, impacts on mental health, and overall quality of life were asked in wave six using a 5-point Likert-type scale, including options such as mental health being much worse during COVID-19, somewhat worse, about the same during COVID-19, somewhat better, and much better during COVID-19 (Supplementary Materials). Wave five also featured the open-ended question, “What is the biggest struggle that you’re having at this point in time, as a result of (or related to) the coronavirus (COVID-19) pandemic?”

#### 2.2.4. Demographics

Participants across all waves were asked a series of demographic questions including age, gender, annual household income, primary health insurance form, residence type (suburban, urban, rural), highest level of education attained, and employment status. Age was selected from a dropdown menu, while others allowed participants to choose from a categorical list of item responses. Participants were asked to select chronic health conditions from a list including, but not limited to, COPD, migraine, asthma, HIV, rheumatoid arthritis, hypertension, and several types of cancers (Supplementary Materials).

#### 2.2.5. Analysis

Responses were analyzed using descriptive statistics and z-tests to explore differences across waves. This analysis was conducted in order to identify differences in the need for mental and/or emotional support and changes to quality of life relative to each wave of the survey and the corresponding time period of the pandemic. Responses to open-ended questions were reviewed for common themes and impactful quotes regarding the need for social support.

## 3. Results

### 3.1. Participants

In total, there were 10,177 responses across all six waves. The number of survey participants for each wave is shown in Table 1. Demographics of participants for each wave, including, but not limited to, mean age, gender, and employment status, were collected (Table 2). The most commonly experienced chronic health condition was hypertension, followed by asthma, migraine, rheumatoid arthritis, COPD, and multiple sclerosis. Nearly seven in ten had never been diagnosed with cancer. Of those diagnosed with cancer, skin cancer (of any form) was the most common.

The majority of participants were female; however, this is in line with prior research among people who were seeking health information or support online [30]. Unsurprisingly, given the chronically ill nature of participants and the older average age, nearly half were either on disability benefits or fully retired, and roughly 30% were either employed full-time, part-time, or self-employed (Table 2).

### 3.2. COVID-19 Concern

The percentage of respondents reporting a 6 or 7 on the scale of general COVID-19 concern stayed relatively constant throughout waves one through five, peaking in the first wave with 71% of respondents (*n* = 699) and remaining in the mid-to-high 60% range through to wave five. In wave six, the question was adjusted slightly to inquire about the concern of contracting COVID-19 (after over one year of infections and the introduction of several vaccines). As such, significant concern for personally contracting COVID-19 in wave six was only 38% (*n* = 370). The trends in patterns of concern with regard to personal health history were the opposite for those with general chronic health conditions versus those with cancer. Those with a cancer history reported the highest levels of concern for COVID-19 in relation to their personal health at the beginning of the pandemic (wave one, 86%, *n* = 105). However, this significantly decreased to the 40% range for waves two through four (*p* < 0.01). In contrast, those with non-cancerous chronic health conditions saw a steady trend in concern for COVID-19 in relation to their personal medical history, with those reporting a strong concern hovering between 67% and 71% throughout the field of study.

### 3.3. Self-Directed Research and Desire for Additional Support

The top sources respondents used for COVID-19-related information throughout waves one through four were TV news reports (64%, *n* = 4591), government websites such as the CDC or NIH (61%, *n* = 4393), news websites (57%, *n* = 4099), and Internet search engines such as Yahoo or Google (47%, *n* = 3353). Social networking sites such as Facebook and Twitter were used by 38% of respondents (*n* = 2721), and online blogs and support communities were used by 17% (*n* = 1196). Internet searches, websites for healthcare professionals such as academic journals, TV news reports, and social media were all utilized more heavily in the beginning of the pandemic relative to later waves (*p* < 0.01 for wave one versus waves two, three, and four, individually). Online blogs and support communities were utilized significantly more in wave two (19%, *n* = 426, *p* < 0.01) than in any other wave, coinciding with peak lockdown times in the United States (April 2020).

When asked which types of information and/or support would be most helpful to respondents, the three most commonly chosen sources were up-to-date and accurate information about COVID-19 (47%, *n* = 2921), emotional and/or mental health support (25%, *n =* 1581), and financial support for other necessities/bills (22%, *n* = 1356). Most notably, the desire for emotional and/or mental support was highest in the final wave—wave four (*p* = 0.01 compared to wave two and *p* < 0.01 for wave three).

### 3.4. Burnout, Isolation, and Mental Health Distress

The impacts on mental and emotional health were assessed most directly in waves five and six, after roughly a year of the COVID-19 pandemic. Nearly 60% (*n* = 1143) of respondents in wave five said the pandemic had increased the level of stress and/or anxiety in their daily life, compared to 9% (*n* = 183) who did not share this perception (*p* < 0.01), and 62% (*n* = 1240) were currently worried about returning to “normal” activities (*p* < 0.01). Additionally, in wave five, when asked about family and friends, 38% (*n* = 764) reported that they are keeping in touch with loved ones less than before the pandemic, compared to only a quarter (*n* = 500) reporting an increase in connection (*p* < 0.01). Notably, about one quarter of those who sought telehealth for any reason in this group did so for a mental health counseling or therapy session (25%, *n* = 371).

In wave six, 60% (*n* = 588) said quality of life was somewhat or much worse as a result of the pandemic, while only 9% (*n* = 86) reported an improvement (*p* < 0.01). When asked directly about mental health impacts, 56% of this group felt their mental health was worse or much worse during the pandemic compared to pre-pandemic (*n* = 547, *p* < 0.01), and only 6% (*n* = 63) reported improvements in mental health.

### 3.5. One-Word Perceptions

**Figure 1:**
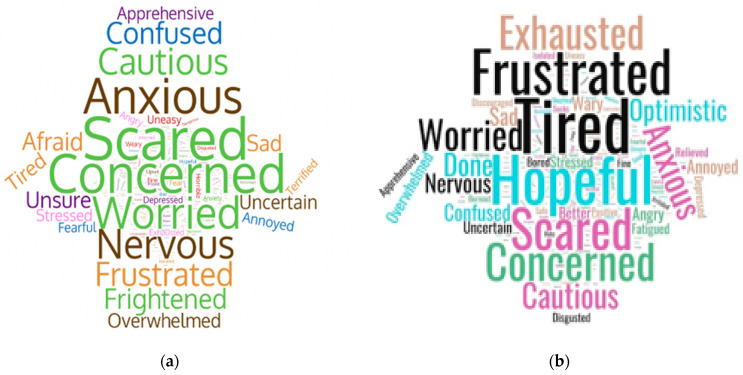
One-word perceptions of the pandemic in word-cloud format: (**a**) Wave four (July 2020); (**b**) Wave five (April 2021).

Responses to the one-word, open-ended question about current feelings regarding COVID-19 were reviewed and a word cloud figure was generated (Figure 1). In the word cloud format, the most commonly cited words are represented as larger in the figure. As shown in Figure 1, earlier in the pandemic, aligned with lockdowns and CDC calls to practice social distancing, the terms “anxious”, “scared”, and “concerned’ were the most frequently cited. In wave six, more than a year after the pandemic began and with vaccines becoming available, words like “frustrated”, and “tired” are still prominent; however, new terms like “hopeful”, “optimistic”, and “cautious” are entering into the vocabulary again, occupying more prominent positions in the shared consciousness.

### 3.6. First-Hand Accounts of Isolation and Longing for Connection

Wave five included an additional open-ended response where respondents were asked about their biggest current struggle in relation to the COVID-19 pandemic. Upon the first review of responses, isolation, stress, mental health concerns, and longing for social connectedness were common themes. Several notable responses included:

*“The biggest struggle for me is the isolation. The last time I was out to eat with friends or shopping in a store was the end of February 2020. It’s a more mental/emotional struggle most days.”*

*“Boredom due to seclusion. Normally I busy myself with helping others but being secluded in my room with little to do has begun to wear down my normally positive attitude.”*

*“I don’t have any help at home, and it’s hard for me to manage. I feel incredibly isolated-which causes increased depression. I cannot even participate in communal worship because of immunosuppressant medications that increase my risk for COVID. I would like to work, as much as I can, but as a piano teacher, it is not possible, and that is more isolating and makes one feel more ‘useless.’ Being ill, unemployed, and having no one to have physical contact or interactions with is not normal. It is not conducive to mental health, [which is] hard enough for those with chronic pain and health conditions.”*

Alongside survey responses, Health Union’s OHCs were continuously monitored for trends in member engagement. Notably, concurrent with the start of the survey fielding in late March 2020 (and the initial increasing mandates to stay at home due to the COVID-19 pandemic), the following unprompted response was shared Health Union’s OHC dedicated to asthma:

*“Warm greetings to everyone in the asthma world! As I sit here on day 22 of isolation during the COVID-19 pandemic, I am reminded of my reasons for wanting to share my experiences as an asthma patient and a lung cancer survivor, to name a couple of my health issues. So I am in isolation and I can’t help but think about how grateful I am to have this forum to turn to. Not just in today’s current environment but always. It is so helpful to hear from so many others who are in the same boat. Asthma often makes me feel isolated and alone. In reality, I am alone, but WE are together in our little corners of the planet doing our best to stay safe and healthy and live our lives. The current climate of the world has intensified that for us.”*

## 4. Discussion

The overall objective of this study was to determine OHC members’ changing perceptions of the COVID-19 virus and its impact on mental wellbeing and need for emotional or social support, as well as to determine where people are seeking their information and support needs from. Although government websites, TV news reports, and Internet searches dominated as information sources early in the pandemic, online blogs and support communities (including OHCs) reached their peak reported usage during the time of mass lockdowns and uncertainty in the U.S. (April 2020). This difference in the time of the pandemic suggests that people living with chronic health conditions sought social connectedness, validation, and peer-to-peer information in a time when they were experiencing more distress. This notion was further strengthened by the overall increasing desire for emotional and/or mental support as the pandemic progressed from the time of waves two through four. 

While TV news sites, general Internet searches, and need for financial support were always of high priority to respondents no matter the time frame, the steadily increasing interest in OHCs speaks to the desire for human connection and social support from others during an unpredictable time. By providing a safe and always available online space for these connections, OHCs can provide trusted information, validate concerns and emotions, and provide social support in order to enhance wellbeing.

General concern for COVID-19, specifically concern about the risk of being infected, decreased by wave six when a multitude of infections in the U.S. had already occurred (including potentially among respondents or their family members, thereby potentially decreasing their fear of the unknown). “COVID fatigue” was at an all-time high according to public opinion polls and news reports [32], and several vaccines were newly granted emergency use authorization by the FDA and were becoming widely available. The concerns associated with the earlier phases of the pandemic, such as fear of the unknown and concern about becoming infected, were replaced by new concerns. These included burnout, negative quality of life impacts, reduced connection to loved ones, and mental health deterioration. A large majority reported that the pandemic increased the stress and anxiety they felt in their daily lives, and that they were still apprehensive about returning to a pre-pandemic “normal”. 

During this same time period, although daily COVID-19 concerns may have been subdued, these were replaced with mental and emotional exhaustion. Despite these high levels of distress, survey participants indicated that they did not engage with formal mental health services. In spite of the high overall use of telehealth services in wave five (73%), only a quarter of these visits were for mental health concerns. This suggests that although mental health issues and the need for support rose throughout the pandemic, there are still barriers (whether they are social, financial, practical, or physical) to being able to access mental health services. From a socialization standpoint, OHCs may be able to facilitate trust among users of those communities and help normalize discussions around how to seek mental or emotional support, as well as provide encouragement and validation to others who may be uncertain about using telehealth for mental health services. 

Based on responses to the one-word perceptions of COVID-19 and the first-hand accounts of isolation shared in response to open-ended questions, people with chronic health conditions are willing and ready to discuss these sensitive topics in OHCs. By continuing to utilize the Adaptive Engagement Model, conversations around emotional and mental health can be validated, and encouraged within a safe and supportive online environment. 

OHCs are not without their own inherent risks, however, as shown in recent news accounts of the failure of leading social media companies to appropriately respond to negative impacts [33]. Personal attacks and factually incorrect medical information may arise, and depend on individual members to think critically about information presented and separate personal affiliation from safety [25,34,35]. This can be especially dangerous when it comes to sensitive discussions around emotional and mental health topics, and mentions of potential self-harm. In order to combat this, some OHCs, such as those operated by Health Union, utilized trained moderators, wrote community rules that were shared and enforced through moderation practices, and modeled appropriate responses to users of the OHC. Moderators may have a background in health or social services, and include experienced patient advocates and trained employees who monitor for safe discourse and provide conversation encouragement, resources, and validation where appropriate.

As mentioned above, there is also the risk for mentions of self-harm when creating open dialogue around sensitive and emotional issues. One aspect of OHCs is the unpredictability of people sharing comments or concerns that may not be germane to shared content or topics, and this is often the case when people mention mental health or emotional health concerns. As the COVID-19 pandemic shows in stark relief, existing OHCs, organized around health topics, may quickly become the source that people turn to when seeking support from others. Being prepared for such conversations, with both a strategy as well as an experienced and trained group of community moderators, is critical to quickly adapt to the demands of the pandemic and similar public health crises.

### Limitations

There are several limitations to this study. First, in order to adapt to the changing nature of the COVID-19 pandemic while keeping surveys manageable for chronically ill respondents, several questions from the first survey were removed or tailored across subsequent waves. This made it impossible to compare responses to all variables across all six waves; however, the sample sizes for each survey wave were large enough to gain an understanding of data points of interest from smaller wave groups. Additionally, participants were recruited from people who visited one of the 28 OHCs, clicked on a survey advertisement, and proceeded to the survey consent page, or who had previously opted-in to receive email communications. This recruitment method may have led to sampling bias, specifically for those who had already participated in an OHC environment. Further, although the survey was designed to be as user-friendly as possible, it was not completed by all participants which may indicate that those who completed the survey are more comfortable with the online survey technology, and thus, OHCs. 

Future research would be best served by investigating need for mental and/or emotional health support at this current juncture in the pandemic, and by utilizing similar or identical questions throughout all iterations that further explore why people choose to come to (or avoid) OHCs, what information they may be looking for, how they find support or give support to others within these spaces, and if they feel empowered to seek further help from a healthcare professional when needed.

## 5. Conclusions

Along with all of society, individuals with chronic illnesses fully experience the impacts of the COVID-19 pandemic, and are likely and are likely to seek out information early and avail themselves of risk reduction strategies. Changes in desired resources over time show an increasing interest from basic information about the virus, transmission risk, and ways to minimize risk, to seeking emotional support. The interest and willingness to talk about mental health impacts and the need for support speaks to the desire for human connectedness in times of societal upheaval and the resultant severe isolation. 

The literature reviewed provides a backdrop for the current study and illustrates issues relevant to social connectedness for people with chronic health conditions. This includes the potential negative impact of social media on mental health, which has salience for people with chronic health conditions, particularly during the pandemic. While the increase in the availability of telehealth during the pandemic has the potential to increase the use of mental health services, it is less clear that people were able to avail themselves of these opportunities. 

The first-hand accounts of people living with chronic health conditions struggling to find support and social contact are illustrated in the responses to open-ended questions and show a range of emotional impacts and coping strategies. Although mental and emotional distress are common, not all who need professional support feel comfortable or know how to access those services. 

OHCs can provide an intermediate and safe space where conversations about mental and emotional wellbeing can be normalized and those in need are encouraged to seek additional assistance from healthcare professionals if warranted. However, OHCs cannot do this through passive engagement only. Active moderation of OHCs using trained and experienced moderators can provide a safe space with planned, real-time strategies to address crisis situations, including future pandemics or public health emergencies, as they arise. This research supports the thesis that OHCs, when managed and moderated appropriately, have the power to normalize mental health discussions, thus providing a unique value to those who experience mental health concerns.

## Figures and Tables

**Table 1:** Survey wave sample size, number of OHCs included, dates fielded, and completed responses.

Survey Wave	Dates Fielded	Num. of OHCs	Total Completes
Wave 1	19–25 March 2020	10	991
Wave 2	14–17 April 2020	26	2214
Wave 3	12–14 May 2020	26	2210
Wave 4	21–23 July 2020	26	1777
Wave 5	23 October–2 November 2020	26	2005
Wave 6	12–19 April 2021	28	980

**Table 2:** Survey participants; select demographics per wave.

Survey Wave	Gender	Mean Age	Employment Status
Wave 1	87% Female 12% Male	58.7	35% Employed 34% Retired 21% Disability
Wave 2	84% Female 16% Male	57.6	33% Employed 30% Retired 24% Disability
Wave 3	81% Female 19% Male	56.3	30% Employed 38% Retired 21% Disability
Wave 4	83% Female 17% Male	59.7	31% Employed 36% Retired 22% Disability
Wave 5	82% Female 18% Male	58.9	30% Employed 38% Retired 21% Disability
Wave 6	78% Female 21% Male	60.4	31% Employed 39% Retired 19% Disability

## Data Availability

The data presented in this study are available by request from the corresponding authors. Additional summary reports using Data from the surveys described in this study are available here: https://health-union.com/blog/covid-19-resources-pharma-marketing (accessed on 30 November 2021).

## Supplementary Materials

The following are available online at https://www.mdpi.com/article/10.3390/ijerph182312743/s1, File S1: Wave 5 Survey Questions.
