# Supplementary material for: Come for Information, Stay for Support: Harnessing the Power of Online Health Communities for Social Connectedness during the COVID-19 Pandemic
Source: Int J Environ Res Public Health. 2021 Dec 3;18(23):12743. doi: 10.3390/ijerph182312743 (PMC8656945; doi:10.3390/ijerph182312743)
Supplement: Supplementary file 1 [file ijerph-18-12743-s001.zip › ijerph-1463596-supplementary.pdf]

File S1: Wave 5 Survey Questions

Novel Coronavirus: How are Consumers Responding – Wave 5

8-10 minute online survey

Health Union (parent company of sites like MultipleSclerosis.net, Asthma.net, and others) is conducting a survey to **better understand the perspectives** of people living with **chronic health conditions** on the **novel coronavirus (COVID-19)**.

This survey should take approximately **8-10 minutes to complete**. As a thank you, if you qualify and complete, you will be entered into a drawing to win **one of 100 $25 e-gift cards**.

Since we are looking for specific people, it is required that a set of questions be answered to determine if you qualify. Not everyone will qualify for this survey - if you do not qualify you will not be able to take the remainder of the survey and will not be eligible to win one of the e-gift cards. Please note that this does not impact your participation or qualification in future surveys.

If you meet the criteria and complete the survey, you will be entered into a drawing to win a $25 e-gift card in exchange for your participation. Winners will be notified via email within 10 business days after the survey closes.

- The survey should take approximately 8-10 minutes to complete
- Participation in this survey is limited to a first-come, first-served basis
- We will leave the survey open until we have the required number of participants
- Participation in this survey is completely optional
- The survey is anonymous, and all results will be analyzed in aggregate (eg, 60% of people said that...)

You may only take this survey once. Surveys detected as having been taken more than once or fraudulently by an individual and/or entity will not be eligible to win a e-gift card.

The terms and conditions for the e-gift card giveaway can be found here.

Please note that once you page forward in the survey, you **may not be able go back**. Take a moment to read the content presented to you and review your responses before clicking Next.

Health Union reminds you to never start, stop or change any medication without the advice of your healthcare professional.

1. Would you like to start the screening survey to see if you qualify?
   - ○ Yes, I would like to start the screening survey
   - ○ No thank you TERMINATE NOW

2. What is your age?
   <Drop-down menu with options ranging from Under 18, 18, 19, 20 … to 89, 90 or older>
   TERMINATE NOW if <18

3. Which best describes where you live?
   - ○ I live in the US
   - ○ I do not live in the US TERMINATE NOW

4. Have you been formally diagnosed by a healthcare professional with any of the following conditions? (Select all that apply)
   - ☐ Alzheimer’s disease
   - ☐ Ankylosing spondylitis (AS)
   - ☐ Asthma
   - ☐ Atopic dermatitis/eczema
   - ☐ Axial spondyloarthritis/non-radiographic axial spondyloarthritis
   - ☐ COPD/emphysema/chronic bronchitis
   - ☐ Crohn’s disease
   - ☐ Cystic fibrosis
   - ☐ Endometriosis
   - ☐ Heart failure
   - ☐ Hepatitis C
   - ☐ High blood pressure/hypertension
   - ☐ High cholesterol/hyperlipidemia
   - ☐ HIV
   - ☐ Irritable bowel syndrome (IBS)
   - ☐ Lupus
   - ☐ Macular degeneration
   - ☐ Migraine
   - ☐ Multiple sclerosis (MS)
   - ☐ Parkinson’s disease
   - ☐ Plaque psoriasis
   - ☐ Psoriatic arthritis (PsA)
   - ☐ Rheumatoid arthritis (RA)
   - ☐ Type 2 diabetes
   - ☐ Ulcerative colitis (UC)
   - ○ None of these

ASK if have MIGRAINE

5. **Prior to starting your most recent migraine treatment,** about how many migraine days did you experience during a typical month? ______ [range is 0 – 31]

6. Have you ever been diagnosed with any of the following cancers? (Select all that apply)
   - ☐ Bladder cancer
   - ☐ Blood cancer (eg, leukemia, lymphoma, multiple myeloma, MDS)
   - ☐ Breast cancer
   - ☐ Colon cancer (colorectal cancer)
   - ☐ Head or neck cancer
   - ☐ Kidney cancer (renal cancer)
   - ☐ Lung cancer
   - ☐ Ovarian cancer
   - ☐ Prostate cancer
   - ☐ Skin cancer (eg, melanoma, basal cell carcinoma, squamous cell carcinoma)
   - ☐ Other type of cancer (please specify): _________
   - ☐ I have not been diagnosed with any cancers

SHOW IF BLOOD CANCER SELECTED

7. Which specific blood cancer diagnosis did you receive **most recently**?
   - ○ A type of leukemia (eg, ALL, AML, CLL, CML)
   - ○ A type of lymphoma (eg, Hodgkin lymphoma, Non-Hodgkin Lymphoma)
   - ○ Multiple myeloma
   - ○ Myelodysplastic syndromes (MDS)
   - ○ Myeloproliferative neoplasms (MPNs, eg, polycythemia vera, myelofibrosis, essential thrombocythemia)
   - ○ Other type of blood cancer

SHOW IF SELECT BLADDER, BREAST, LUNG, OVARIAN, PROSTATE, SKIN CANCER

8. What type/stage of cancer has your doctor told you that you have **most recently**?
   - ○ Early stage (Stage I or II, or limited stage)
   - ○ Late stage (Stage III – not metastatic)
   - ○ Metastatic (Stage IV or extensive stage)
   - ○ Currently in remission (or no evidence of disease)
   - ○ Don't know/Not sure

MUST SELECT 1 HU Chronic Health Condition or 1 HU Cancer that we have a community for (bladder, blood, breast, lung, prostate, skin)- ELSE TERMINATE NOW

9. Have you heard about the novel coronavirus (COVID-19)?
   - ○ Yes
   - ○ No TERMINATE – Keep track of count in tracking

Survey Intro

You meet the criteria for this survey! If you complete this survey in its entirety, you will be entered to win one of 100 $25 e-gift cards.

10. At this time, how concerned do you feel about the novel coronavirus (COVID-19)?

Not at all concerned						Very concerned
1	2	3	4	5	6	7

11. What is the biggest struggle that you're having at this point in time, as a result of (or related to) the coronavirus (COVID-19) pandemic? ____

© 2021 Health Union, LLC

12. We're interested in how the coronavirus pandemic may have changed some of your habits or behaviors. Compared to how often you did the following before the pandemic, about how often do **you do them now?**

RANDOMIZE	Doing LESS than before the pandemic	About the SAME as before the pandemic	Doing MORE than before the pandemic
Look up information online about my condition	○	○	○
Look up information online about medications or treatments	○	○	○
Keep in touch with friends and family	○	○	○
Use social media	○	○	○
Read/watch the news online or on TV	○	○	○

13. In what ways, if at all, are you "returning to normal" (ie, resuming activities you may have stopped because of the coronavirus pandemic)? (Select all that apply)
Please check an item if you have started doing it – even in a different way and/or with precautions

- ☐ No changes – I am **not** "returning to normal" at this time
- ☐ Attending large gatherings
- ☐ Less "social distancing" (eg, starting to shake hands, give hugs, close personal contact, etc)
- ☐ Going out in public more (eg, movies, restaurants, etc)
- ☐ Returning to my place of work (ie, not working from home anymore)
- ☐ Starting any medication I "paused," skipped, or stopped
- ☐ Planning trips/vacations
- ☐ Stop wearing a mask/protective equipment in public
- ☐ Returning to in-person doctor/healthcare visits
- ☐ Visiting/hanging out with friends/family in person
- ☐ Making more "non-essential" shopping trips
- ☐ Being less vigilant about cleaning/sanitizing my home/items
- ☐ Going to hair salon, barber, and/or nail salon
- ☐ Other (specify _____)

© 2021 Health Union, LLC

14. Since the coronavirus pandemic began (February/March 2020), how many of the following types of visits have you had with a doctor/healthcare professional?

Telehealth is a virtual visit with a healthcare professional through video conferencing and/or phone conversation. This can be an alternative way of having an in-person office visit.

Telehealth visit	In-person office visit
○ None	○ None
○ 1	○ 1
○ 2	○ 2
○ 3	○ 3
○ 4	○ 4
○ 5 or more	○ 5 or more

ASK IF HAD 1 OR MORE OF TYPE OF APPT

15. You indicated you had the following types of doctor/healthcare professional visits **during the pandemic**. What were these visits for? (Select all that apply)

ONLY SHOW COLUMNS FOR TYPES OF VISITS THEY HAD>>>	Telehealth visit	In-person office visit
Routine/periodic visit (eg, annual visit, planned 'check-in' visit)	☐	☐
Illness (flu/cold symptoms, urinary tract infection, etc)	☐	☐
Monitoring for COVID-19 symptoms/what to do if experiencing COVID-19 symptoms	☐	☐
Being newly diagnosed with or seeking a diagnosis of a health condition	☐	☐
New or worsening symptoms related to my health condition(s)	☐	☐
Evaluating if a new medication or treatment is working	☐	☐
Prescription refills of a current medication	☐	☐
Review of results of blood or medical tests (eg, CT scan, MRI)	☐	☐
Mental health counseling or therapy session	☐	☐
Screening to determine if an in-person visit was needed	☐	☐
Other	☐	☐

© 2021 Health Union, LLC CONFIDENTIAL & PROPRIETARY

ASK IF HAD 1 OR MORE OF TYPE OF APPT

16. Were any of the following discussed during one of your doctor/healthcare professional visits **during the pandemic**? (Select all that apply)

ONLY SHOW COLUMNS FOR TYPES OF VISITS THEY HAD>>>	Telehealth visit	In-person office visit
**Started** or added a new medication to my treatment plan	☐	☐
**Switched** from my medication at the time to a different medication	☐	☐
**Stopped** using a medication I was taking (and did not start a new one in its place)	☐	☐
**Stayed** on my current medication as prescribed	☐	☐
**Changed the dosage** of a current medication	☐	☐
Discussed medication side effects	☐	☐
None of the above	☐	☐

ASK if STARTED MEDS

17. You mentioned that you and your doctor/healthcare professional discussed **starting or adding** a new medication to your treatment plan during the pandemic. Which of your health conditions was this for? (Select all that apply)
    - ☐ CARRY FORWARD HEALTH CONDITIONS SELECTED
    - ☐ Another health condition (please specify):_______

SHOW STATEMENT AND ASK BELOW 2 QUESTIONS ON SAME SCREEN IF DISCUSSED “Switching to a new medication” with HCP

You mentioned that you and your doctor/healthcare professional discussed **switching medications** during the pandemic.

18. Which of your health conditions was this for? (Select all that apply)
    - ☐ CARRY FORWARD HEALTH CONDITIONS SELECTED
    - ☐ Another health condition (please specify):_______

19. What was the **primary reason** for this change?
    - o The medication wasn’t working well enough to manage my condition/symptoms
    - o My condition worsened/progressed or I was experiencing new symptoms
    - o I was experiencing side effects and needed to change medications
    - o I could no longer afford the medication and needed to replace with one that I could afford
    - o I was having issues getting access to this medication due to COVID-19
    - o Other reason (not listed)

ASK if DISCUSSED “Staying on my current medication as prescribed”

20. You mentioned that you and your doctor/healthcare professional discussed **staying on your current medication** during the pandemic. What was the primary reason?
    - o My condition was stable/no progression or worsening
    - o We decided to continue monitoring my condition and discuss a possible change at another time
    - o My healthcare professional didn’t want me to make a change during the pandemic
    - o I didn’t want to make a change during the pandemic
    - o My healthcare professional didn’t want me to make a change for a reason other than the pandemic

- I didn’t want to make a change for a reason other than the pandemic
- My healthcare professional didn’t want to make a change without an in-person visit – SHOW if Have Telehealth
- I didn’t want to make a change without an in-person visit – SHOW if Have Telehealth
- Other reason (not listed)

ASK IF HAD AT LEAST 1 TELEHEALTH VISIT

21. Below are a few different aspects of a typical visit with your doctor/healthcare professional. Please rate how your **most recent telehealth visit** compared to in-person office visits on these aspects.

RANDOMIZE	**Much worse** than in-person office visits		**About the same** as in-person office visits		**Much better** than in-person office visits
Convenience	○	○	○	○	○
Amount of time spent with the doctor	○	○	○	○	○
Quality of conversation	○	○	○	○	○
Thoroughness of examination	○	○	○	○	○
Ability to ask all of my questions/voice my concerns	○	○	○	○	○

22. Some people view telehealth as acceptable (or even prefer it) for some types of doctor/healthcare professional visits, but consider it less appropriate for other types of visits.

For each type of visit and/or topic of discussion below, please indicate whether you would prefer that it take place via telehealth, whether you’d prefer it to be in-person, or if either is fine.

Telehealth is a virtual visit with a healthcare professional through video conferencing and/or phone conversation. This can be an alternative way of having an in-person office visit.

	Prefer Telehealth Visit	Either is fine	Prefer In-Person Visit
Annual check-up/primary care visit	○	○	○
Routine/periodic check-up visit for my health condition(s)	○	○	○
Discussing new symptoms of my current health condition(s)	○	○	○
Starting a new medication or treatment	○	○	○
Discussing a change in medication or treatment	○	○	○
Evaluating if a new medication or treatment is working	○	○	○
Unplanned visits/discussions because of worsening/progression/flare ups of current symptoms/condition	○	○	○

IF HAD A TELEHEALTH or HCP VISIT

23. Please rate your agreement with the statements below about your experience with your doctor/healthcare professional **since the start of the pandemic.**

RANDOMIZE	Completely disagree						Completely agree
I had a positive experience during my telehealth visit – IF HAD TELEHEALTH VISIT	1	2	3	4	5	6	7
The telehealth video technology was difficult to use – IF HAD TELEHEALTH VISIT	1	2	3	4	5	6	7
My doctor/healthcare professional's office took appropriate precautions during my in-person visit – IF HAD IN-PERSON	1	2	3	4	5	6	7
I wish I could have had a telehealth visit instead of the in-person visit – IF HAD IN-PERSON	1	2	3	4	5	6	7
I felt safe going to my doctor/healthcare professional's office – IF HAD IN-PERSON	1	2	3	4	5	6	7

24. How likely are you to consider using telehealth in the future even after the coronavirus pandemic is more under control?

Not at all likely to consider 1	2	3	4	5	6	Extremely likely to consider 7
○	○	○	○	○	○	○

ASK if DID NOT HAVE AN IN-PERSON HCP VISIT

25. You indicated that you have not had an **in-person office visit** with a healthcare professional since the start of the pandemic. Which, if any, of the following are reasons why you have not had an in-person office visit? (Select all that apply)

RANDOMIZE – Let them multi-select all the options

- ☐ It's not time for my periodic/routine visit yet
- ☐ My healthcare professional(s) office has been closed during the pandemic
- ☐ My visit was cancelled/postponed (by me or my doctor) due to the pandemic
- ☐ My visit was cancelled/postponed (by me or my doctor) due to a reason other than the pandemic
- ☐ My healthcare professional wanted to see me via telehealth
- ☐ I preferred to see my healthcare professional via telehealth
- ☐ I don't feel comfortable going in-person right now
- ☐ I cannot afford the cost/I no longer have health insurance to cover the cost of the visit
- ☐ Other (please specify):___
- ☐ I haven't needed to visit with a doctor/healthcare professional (eg, my condition/symptoms are managed)

© 2021 Health Union, LLC CONFIDENTIAL & PROPRIETARY

ASK if DID NOT HAVE A TELEHEALTH HCP VISIT

26. You indicated that you have not had a **telehealth visit** with a healthcare professional since the start of the pandemic. Which, if any, of the following are reasons why you have not had a **telehealth visit**? (Select all that apply)

    RANDOMIZE - Let them multi-select all the options

    - ☐ It’s not time for my periodic/routine visit yet
    - ☐ My healthcare professional(s) does not offer telehealth
    - ☐ I prefer to see my healthcare professional in person
    - ☐ My healthcare professional wanted to see me in person
    - ☐ I have a health condition/concern that requires me to be there in person
    - ☐ I don’t know how to set up a telehealth visit
    - ☐ I have concerns about the privacy/security of my personal information
    - ☐ The technology is hard to use
    - ☐ I do not have reliable access to the Internet/technology needed
    - ☐ My health insurance does not cover the cost of these visits/I cannot afford the cost
    - ☐ I do not want to use telehealth
    - ☐ Other (please specify):___
    - ☐ I haven’t needed to visit with a doctor/healthcare professional (eg, my condition/symptoms are managed)

27. So far, how much of a negative impact has the novel coronavirus (COVID-19) had on your finances?

**No negative impact**						**Significant negative impact**
1	2	3	4	5	6	7

28. Since the start of the pandemic, which of the following have you personally experienced? (Select all that apply)

    - ☐ I have lost my job (employer’s choice)
    - ☐ I have quit my job to be a caregiver to my children and/or parents (my choice)
    - ☐ I have quit my job for another reason
    - ☐ I have reduced the number of hours I work
    - ☐ I have experienced a reduction in salary or wages
    - ☐ I have started working from home
    - ☐ I have taken a medical leave from work
    - ☐ I lost my health insurance coverage
    - ☐ I took time off from college/school
    - ☐ I stopped taking a medication because of its cost
    - ☐ I started skipping/stretching out doses of medication because of its cost
    - ☐ I used an over-the-counter medication **instead of using** a prescription medication
    - ☐ I struggle to pay for healthcare costs (doctors’ appointments, medications)
    - ☐ I struggle to pay for household expenses (rent/mortgage, food/groceries, bills)
    - ☐ None of the above

ASK NEXT 2 QUESTIONS ON SAME PAGE

29. Have you been tested for the novel coronavirus (COVID-19)?
    - o Yes, and I tested positive
    - o Yes, and I tested negative
    - o No, I was not tested

30. Have you had an antibody test (blood test) for the novel coronavirus (COVID-19)?
    - o Yes, and I have the antibody
    - o Yes, and I do NOT have the antibody
    - o No, I was not tested

31. Please rate your agreement – **from your point of view –** with the following statements below about the novel coronavirus (COVID-19):

RANDOMIZE	**Completely disagree**						**Completely agree**
I feel like people are not taking the coronavirus seriously enough	1	2	3	4	5	6	7
I feel like I am taking all the right precautions to reduce my risk of getting coronavirus	1	2	3	4	5	6	7
I feel burnt out from the pandemic and its effects/restrictions	1	2	3	4	5	6	7
The pandemic has increased the level of stress and/or anxiety in my daily life	1	2	3	4	5	6	7
I'm hesitant to go to medical facilities (eg, doctor's office, urgent care, hospital) because of the pandemic	1	2	3	4	5	6	7
I am worried about resuming "normal" activities at this time	1	2	3	4	5	6	7
I sometimes feel embarrassed to tell people I had COVID-19 – ASK if TESTED POSITIVE FOR VIRUS	1	2	3	4	5	6	7
When I tested positive, I feel like the doctors/nurses gave me all the information I needed about what COVID-19 is and what it meant for me – ASK if TESTED POSITIVE FOR VIRUS	1	2	3	4	5	6	7

We just have a few final questions to learn a little bit more about you.

32. IF DO NOT HAVE CANCER: Which, if any, of the following **prescription** treatments **are you currently using** to manage your condition(s)? (Select all that apply)

IF HAVE CANCER: Which, if any, of the following **prescription** treatments **are you currently using** to manage your condition(s) and/or receiving to treat your cancer? (Select all that apply)

Display if have an autoimmune condition: *Please note that the phrase "autoimmune condition" refers to the following health conditions: Ankylosing spondylitis (AS), Atopic dermatitis, Axial spondyloarthritis/non-radiographic axial spondyloarthritis, Crohn's disease, Lupus, Plaque psoriasis, Psoriatic arthritis (PsA), Rheumatoid arthritis (RA), and Ulcerative colitis (UC)*

Only show medications related to their condition/cancer – Headers is ONLY for HU use

- ☐ Anti-depressant and/or anti-anxiety medications (eg, Cymbalta (duloxetine), Elavil (amitriptyline), Effexor (venlafaxine)

Asthma/COPD (/emphysema/chronic bronchitis)

- ☐ Short acting/rescue inhaler (eg, ProAir, albuterol)
- ☐ Long acting/maintenance inhaler (taken on a regular basis) (eg, Advair, Symbicort, Incruse Ellipta, etc)
- ☐ Biologic/injectable medication for asthma (eg, Xolair, Nucala, Fasenra, Dupixent, Cinqair)

Migraine

- ☐ Oral **acute/rescue** migraine medication (Taken at first sign of your migraine or when symptoms begin – eg, triptans (such as Imitrex, Frova, Relpax), "gepants" (eg, Ubrelvy, NurtecODT), prescription NSAIDs (such as Aleve, Cambia, and Toradol), or muscle relaxants (such as Valium, Zanaflex, or Flexeril))
- ☐ Oral medication for migraine **prevention** (eg, topiramate, antiepileptic drugs, beta blockers, calcium channel blockers
- ☐ Botox for Chronic Migraine (onabotulinumtoxinA)
- ☐ Injectable CGRP for migraine prevention (eg, Aimovig, Ajovy, Emgality, Vyepti)

Multiple Sclerosis

- ☐ Oral disease-modifying therapy (DMT) (eg, Tecfidera, Aubagio, Gilenya)
- ☐ Injectable disease-modifying therapy (DMT) (eg, Copaxone, Rebif)
- ☐ Infusion disease-modifying therapy (DMT) (eg, Ocrevus, Lemtrada, Tysabri)

Autoimmune conditions (See list above)

- ☐ Topical prescription treatments for psoriasis/atopic dermatitis/skin symptoms (like creams or ointments, eg, topical corticosteroids, Eucrisa, Taclonex, etc)
- ☐ Plaquenil (hydroxychloroquine sulfate)
- ☐ Oral prescription treatments for autoimmune condition **other than Plaquenil/ hydroxychloroquine** (eg, oral corticosteroids, methotrexate, mesalamine, sulfasalazine, Otezla, Xeljanz, etc)
- ☐ Biologic/injectable medication for autoimmune condition (eg, Benlysta, Cosentyx, Enbrel, Dupixent, Humira, Orencia, Remicade, Stelara, etc)

Treatment for cancer

- ☐ Topical treatment for cancer treatment (eg, Efudex, Flouroplex)
- ☐ Radiation therapy for cancer treatment
- ☐ Oral medication for cancer treatment (eg, etoposide, Tarceva, Revlimid, Zejula)
- ☐ Intravesical treatment for cancer (into the bladder - BCG and/or chemotherapy)

- ☐ Injectable medication for cancer treatment (eg, subcutaneous Velcade)
- ☐ IV chemotherapy (eg, Cytoxan, carboplatin, taxotere, Gemzar, etc)
- ☐ IV targeted or immunotherapy for cancer treatment (eg, Keytruda, Opdivo, Imfinzi, Darzalex, Kyprolis, Rituxan)

Treatment for symptoms and effects of cancer

- ☐ Oral medication for cancer symptoms and effects (eg, steroids like prednisone or Decadron, pain medication)
- ☐ Injectable/IV medication for cancer symptoms and effects (eg, Xgeva for bone health, IV steroids)

- ☐ Other prescription medication or treatment (please specify): _____
- o Don’t know/Not sure
- o I am not currently taking a prescription medication and/or on a treatment for cancer [Display end of statement if also have cancer]

D1. In which state do you live? [STATE DROP DOWN]

D2. Which of the following best describes where you live?
- o Rural
- o Urban
- o Suburban

D3. What is your gender?
- o Female
- o Male
- o Non-binary/ Gender non-conforming

D4. What is the highest level of education you have completed?
- o No schooling
- o No schooling past 8th grade
- o Some high school, did not graduate
- o High school graduate, diploma, or the equivalent (eg, GED)
- o Trade/technical/vocational training
- o Some college, no degree
- o 2-year college degree (Associate's degree)
- o 4-year college degree (Bachelor's degree)
- o Master’s degree
- o Doctoral or professional degree (PhD, MD, JD)

D5. What is your annual household income?
- o Less than $30,000
- o $30,000 - $54,999
- o $55,000 - $74,999
- o $75,000 - $99,999
- o $100,000 - $149,999
- o $150,000 - $199,999
- o $200,000 or more
- o Prefer not to answer

D6. What is your current employment status?
- o Employed, full time
- o Employed, part time

- Self-employed
- Unemployed, looking
- Unemployed, not looking
- On disability
- Fully retired
- Student
- Homemaker/Stay-at-home parent

D7. What is your primary health insurance?

- Group coverage, through my employer or the employer of a spouse or family member
- COBRA Insurance
- Private insurance, purchased directly from the insurance company
- Health insurance exchange, enrolled through Affordable Care Act
- Medicare
- Medicaid
- Military coverage (DOD), VA, or TriCare
- Other insurance type/Not sure of what type
- Do not have

33. Do you want to enter into the drawing for one of 100 $25 e-gift cards? If so, please provide your email address: _____

34. Over the next month, we will be conducting phone interviews with respondents from this survey who meet specific criteria. Those selected to participate in the 30-minute phone interview will receive a $25 e-gift card for their participation.

If you meet the criteria for the phone interview, would you like to be contacted for potential participation? By indicating yes, you are not committing yourself, rather someone from our research partner will be contacting you by email to see if you are still interested and able to meet during our research timeframe.

- No, I am not interested
- Yes, please contact me if I meet the criteria (provide contact email): _______

Thank you for taking this survey! We know that there is a lot going on in the world right now – and we hope you are doing as well as can be expected during this time.

If you have any questions about the novel coronavirus (COVID-19), the CDC can be a good resource for information (https://www.coronavirus.gov/) or you can contact your healthcare provider with specific questions.
Health Union reminds you to never start, stop or change any medication without the advice of your healthcare professional.
